# Mood, Disability, and Quality of Life among a Subgroup of Rheumatoid Arthritis Individuals with Experiential Avoidance and Anxiety Sensitivity

**DOI:** 10.1155/2016/7241856

**Published:** 2016-04-17

**Authors:** S. Mehta, D. Rice, S. Janzen, J. E. Pope, M. Harth, A. P. Shapiro, R. W. Teasell

**Affiliations:** ^1^Western University, London, ON, Canada N6A 3K7; ^2^St. Joseph's Health Care London, London, ON, Canada N6A 4V2; ^3^Lawson Health Research Institute, London, ON, Canada N6C 0A7

## Abstract

*Objective.* The current study aimed to identify and characterize distinct RA subgroups based on their level of EA and AS and compares the difference among the subgroups in mood, disability, and quality of life.* Methods.* Individuals with chronic pain for at least 3 months were recruited from an academic rheumatoid clinic. Participants were assessed for demographic, psychosocial, and personality measures. A two-step cluster analysis was conducted to identify distinct subgroups of patients. Differences in clinical outcomes were compared using the Multivariate ANOVA based on cluster membership.* Results.* From a total of 223 participants, three distinct subgroups were formed based on cluster analysis. Cluster 1 (*N* = 78) included those with low levels of both EA and AS. Cluster 2 (*N* = 81) consisted of individuals with moderate levels of EA and low levels AS. Cluster 3 (*N* = 64) included those with moderate levels of EA and high AS. Compared to those in Cluster 1, those in Cluster 3 had significantly higher levels of mood impairment and disability and lower quality of life (*p* < 0.05). Significantly lower levels of mood impairment were seen in Cluster 1 compared to Cluster 2 (*p* < 0.05). However, no significant difference in disability or quality of life was seen between the two groups.* Conclusions.* The three subgroups differed significantly in levels of impairment in mood, disability, and quality of life. However, levels of EA had a greater impact on disability and quality of life than AS.

## 1. Introduction

Rheumatoid arthritis (RA) is an autoimmune disease that causes joint inflammation and leads to bone and cartilage damage [1]. Pain is often a central part of RA, with the majority of RA patients experiencing chronic pain [2]. Chronic pain is a disabling condition that results in considerable suffering and negatively impacts an individual's psychological, social, and economic quality of life (QoL) [3]. Studies examining cognitive behavioural interventions among the RA population have been encouraging in improving treatment effects [4]. However, management of chronic pain can be complex. Even when the disease is well controlled, residual pain is often present.

A variety of psychosocial variables impact coping in chronic pain conditions such as RA [4, 5]. Experiential avoidance (EA) negatively affects pain coping and contributes to increased dysfunction secondary to chronic pain [6]. Experiential voidance is defined as a process whereby there is unwillingness to endure upsetting emotions, thoughts, feelings, and bodily sensations, as well as the circumstances in which they occur [7, 8]. In pain populations, even while controlling for demographic characteristics, pain, acceptance, and mindfulness, EA has been found to significantly predict psychological functioning [9] and is highly correlated with depression, anxiety, stress [10, 11], physical disability, psychosocial disability, and patient function [12]. Furthermore, EA may also be related to negative affective states via anxiety sensitivity (AS) [13]. Anxiety sensitivity is the fear of anxiety-related sensations, specifically, fear of bodily sensations due to beliefs that these sensations will have negative somatic, cognitive, or social consequences [14]. Anxiety sensitivity is often reported to be a vulnerability factor for stress perception.

Both AS and EA are associated with negatively experiencing internal events but they are distinct constructs [15]. Experiential avoidance involves negative private experiences in general, while AS involves arousal-related body sensations specifically. Furthermore, EA is considered more of a psychological process, while AS is considered a trait-like factor [15]. Through its interaction with AS, EA may help to alleviate distress in the short term; however, in the long-term it may exacerbate it. Naragon-Gainey [16] found that individuals who are less likely to stay in contact with unwanted experiences, compared to those that do, perceive their coping resources to be lower. Hence, EA and AS may be important factors in the development of low mood and greater distress.

It has been reported that pain in patients with RA is related to their daily function [17]. Rice et al. (personal communication) found AS was independently associated with stress among RA individuals. However, most evidence regarding the effect of EA and AS is in the chronic pain population while research in the RA population is lacking. Research has found that coping within samples of chronic pain and RA patients are distinct from each other [18]; therefore, examining the role of AS and EA in RA patients is important in determining whether attempts to consciously control thoughts, behaviours, and feelings negatively impact this group of patients and result in poor mood, functioning, and QoL. The aim of the current study was to characterize individuals with RA into clinically relevant subgroups based on their level of EA and AS through a cluster analysis. The secondary objective was to study how the subgroups differed in mood, disability, and QoL. We hypothesized that lower levels of EA and AS would be associated with less disability and greater mood and QoL.

## 2. Methods

### 2.1. Participants

Participants were recruited from an academic Rheumatology Outpatient Clinic, at St. Joseph's Health Care, in London, Ontario, over a 20-month period. Inclusion criteria were as follows: diagnosis of RA by a rheumatologist (using the American College of Rheumatology criteria), persistent pain of greater than 3 on the Visual Analogue Scale, thought to be secondary to RA for greater than three months, and age of 18 years or older. Given that this study involved the completion of self-report questionnaires, those with an inability to read and write English were excluded.

### 2.2. Procedure

Patients who met the inclusion criteria and agreed to participate were referred to the research coordinator by their primary physician. The research coordinator provided potential participants with the letter of information and consent form. Patients were made aware that their decision to participate in the study will in no way interfere with their standard care at the hospital. All patients received individualized pharmacotherapy and psychotherapy as seen fit by the multidisciplinary team. Participants who consented to the study were mailed a questionnaire booklet two weeks prior to their scheduled appointment at the clinic. This mailing was followed by a telephone call from a research assistant who answered the participants' questions and instructed the participants to complete the booklet prior to their appointments. Participants were asked to arrive half an hour before their appointment was scheduled to begin. When participants arrived at the clinic, the research assistant collected the first questionnaire booklet consisting of questions regarding demographics (age, gender, years of education, and relationship status) and then each subject was asked to complete the second booklet of questionnaires containing outcome measures related to personality, coping, disability, pain, and quality of life. The research assistant made sure of clarifying all answers left blank by the participant. This study was approved by the University of Western Ontario Health Sciences Review Board.

### 2.3. Cluster Variable Measures

#### 2.3.1. Anxiety Sensitivity Index (ASI)

The ASI [19] is a 16-item measure of the fear of anxiety-related symptoms comprised of three factors: fear of the somatic symptoms of anxiety, fear of mental incapacitation (“cognitive dyscontrol”), and fear of negative social repercussions of anxiety [20]. Each item is rated on a five-point Likert scale ranging from 0 (very little) to 4 (very much). The instrument's psychometric properties and predictive validity have been well established [19, 21]. We used the total score within our sample as has been previously suggested since studies have found that the subscales are highly correlated, and a greater percentage of items load higher on the general domain factor rather than on the domain-specific factors [22].

#### 2.3.2. Acceptance and Action Questionnaire (AAQ)

The AAQ [23] is a 9-item self-report measure of EA or the unwillingness to remain in contact with distressing private experiences (body sensations, emotions, and thoughts) and the inclination to alter the form or frequency of these experiences. The scale was designed to assess peoples' ability to accept undesirable thoughts and feelings. The 9 items on this scale are answered on a 1-to-7-point scale with lower numbers representing that the item is “never true” while a 7 means that the item is “always true” for the participant. High scores represent EA and low scores reflect acceptance. It yields a single factor solution and is correlated with a wide range of negative behavioural and physical health outcomes [23]. The AAQ demonstrates adequate validity and reliability scores [9].

### 2.4. Outcome Measures

#### 2.4.1. Brief Pain Inventory-Short Form (BPI-S)

The BPI-S [24] is a widely used questionnaire that asks patients to rate their current pain, worst pain, least pain, and average pain on a 10-point numeric scale. Although the BPI-S typically assesses pain in the past 24 hours, instructions were modified so that patients rated their pain over the previous two weeks. Pain ratings for average pain were used in the current study.

#### 2.4.2. Depression Anxiety Stress Scales-Short Form (DASS-SF)

The DASS-SF [25] is a 21-item self-report measure assessing depression, anxiety, and stress over the previous week. This short form scale is an abbreviated version of the 42-item scale developed by P. F. Lovibond and S. H. Lovibond [25] and boasts the same good to excellent psychometric properties as the original scale [26].

#### 2.4.3. Health Assessment Questionnaire- (HAQ-) Disability 

The HAQ-disability and pain scales are self-report measures of function in patients with rheumatic diseases. Disability is assessed by eight categories: (1) dressing, (2) arising, (3) eating, (4) walking, (5) hygiene, (6) reach, (7) grip, and (8) common activities. The HAQ has proved to be a very reliable measure and has been found to predict many features of patients' subsequent disease course [27].

#### 2.4.4. 36-Item Short Form Health Survey (SF-36)

The SF-36 is a 36-item self-report measure that assesses eight domains of health related quality of life. These domains include (1) limitations in physical functioning, (2) social limitations due to emotional or physical troubles, (3) role limitations due to physical health problems, (4) role limitations due to emotional health problems, (5) general mental health, (6) bodily pain, (7) vitality, and (8) general health perceptions [28]. The SF-36 has acceptable psychometric properties [28].

### 2.5. Data Analysis

A two-step cluster analysis was performed using SPSS version 23.0 (Chicago, IL) to organize observations into two or more mutually exclusive groups, where members of the groups shared properties in common. Two clustering variables were used in the analysis: AS and EA based on ASI and AAQ scores, respectively. The log-likelihood distance measure was used to compute likelihood distance between clusters with subjects assigned to the cluster leading to the largest likelihood. Number of clusters was not predetermined. The Bayesian information criterion was used to judge adequacy of the final solution. Differences in sample demographic characteristics were compared according to cluster membership using univariate analysis of variance for continuous variables and *χ* ^2^ tests for categorical variables in order to characterize the resulting clusters. A multivariate analysis of covariance was conducted on outcome measures (DASS-SF, HAQ, and SF-36) according to cluster membership with patient demographic factors including age, gender, pain duration, and pain intensity as covariates. Post hoc analyses were conducted with a Tukey correction. SPSS version 23.0 (Chicago, IL) was used for all tests performed, with the significance level set at 0.05, two-tailed.

## 3. Results

A total of 441 patients were eligible for inclusion within the study, of which 218 refused to participate, leaving a final sample size of 223 patients in the study. The study population consisted of individuals with a mean age of 57.8 years and was predominantly female (75.7%). Almost half of the individuals were employed (48.9%). Thirteen participants (6%) had a diagnosis of comorbid fibromyalgia in addition to RA. Average pain intensity among the individuals was 3.8 on an 11-point scale (0–10) and average pain duration was 14.3 years. Most were married or in a long-term relationship at the time of the study (75.3%; Table 1).

**Figure 1:**
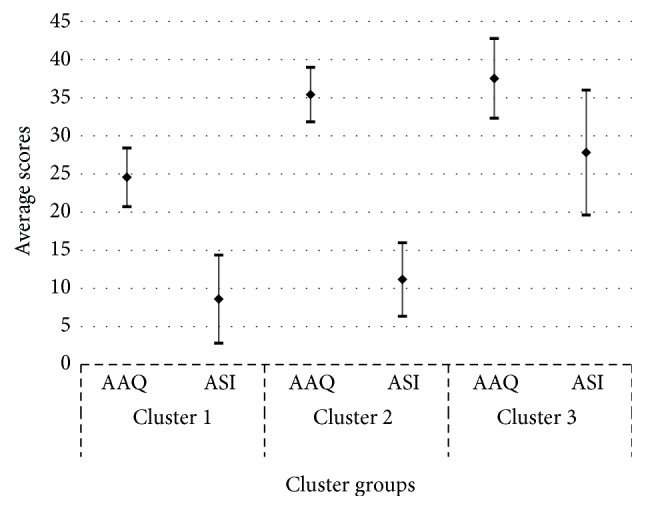
Two-step cluster subgroups based on anxiety sensitivity and experiential avoidance.

The two-step cluster analysis resulted in three subgroups with no exclusion of cases. The clusters were significantly different on both clustering variables. Cluster 1 (*N* = 78) included those with lowest levels of EA (1 SD below mean) and AS (0.75 below mean). These individuals could be best described as adaptive copers. Cluster 2 (*N* = 81) or the average copers included those with levels of EA (0.5 SD above mean) and AS (0.5 SD below mean) who were the closest to the group means. Cluster 3 (*N* = 64) included participants with highest levels of EA (0.75 SD above mean) and AS (1.25 SD above mean); this group represented the dysfunctional copers, see Table 1 and Figure 1. A significant difference was found among the three clusters in employment status (*p* = 0.02). The remaining demographic variables, age, sex, education, relationship status, pain duration, and average pain intensity, were comparable among the three groups (Table 1).

As can be seen in Table 2, a multivariate analysis of covariance with Tukey correction resulted in significant differences among the three clusters in mood, disability, and QoL while adjusting for demographic variables and average pain intensity. Significant differences in DASS-SF total, DASS-SF stress, DASS-SF depression, DASS-SF anxiety, HAQ Total, and SF-36 total scores were seen between adaptive copers and dysfunctional copers (*p* < 0.05), whereby adaptive copers scored significantly higher on mood and QoL and lower on disability compared to dysfunctional copers. Average copers significantly differed from adaptive copers but not from dysfunctional copers on DASS-SF total, DASS-SF stress, and HAQ Total (*p* < 0.05). All groups significantly differed from each other on DASS-SF depression and DASS-SF anxiety (*p* < 0.05), with dysfunctional copers consistently scoring lowest for mood and QoL and highest on disability.

## 4. Discussion

To our knowledge, this is the first study to characterize participants with RA into three subgroups based on their AS and EA levels. The difference in mood, disability, and QoL was also examined among the three clusters: (1) adaptive copers, (2) average copers, and (3) dysfunctional copers, with demographic factors and pain measures as covariates. Consistent with our hypotheses, adaptive copers were more likely to have improved mood, disability, and QoL compared to average and dysfunctional copers. The adaptive copers group was also the only group with EA levels below the average. It may be that even moderate levels of EA may result in impaired mood, disability, and QoL. Even though levels of AS were similar between the adaptive and average copers group, the low levels of EA may act as a protective factor. Adaptive copers may be more accepting of negative bodily sensations and instead of avoiding tasks out of fear of pain they may be persistent in completing them. Those that are adaptive copers may also be less likely to have dysregulation of emotional experiences and life-constricting behaviours that lead to greater disability and lower QoL. Kashdan et al. [8] found an inverse relationship between EA and daily positive emotions, life appraisals, and events. Hence, significantly less issues with mood, disability, and QoL suggest that the adaptive copers may have a more positive outlook in general.

Surprisingly, average copers did not differ significantly from dysfunctional copers on total scores. An explanation for this may be due to the overlap in the level of EA in both subgroups as seen in Figure 1. Therefore, though there was a lack of overlap between the two subgroups in AS, the presence of similarly high levels of EA may have influenced their lack of difference in outcomes. Hence, EA may play a more dominant role in differences in outcomes among individuals with chronic pain secondary to RA. The high levels of EA in both populations may speak to the fact that EA involves a cognitive process that alters the form and frequency of unwanted thoughts and events. It is these thoughts, in turn, that may evoke feelings of limited control, distress, and discouragement, as opposed to physiological sensations of pain intensity. In essence, when avoidance is rigidly applied as a coping strategy, the effort, energy, and attention directed toward controlling the unwanted sensations may paradoxically increase the frequency of these events and lead to even greater dysfunction and distress [23, 29, 30]. Results from the current study are consistent with previous research that using EA as a coping mechanism following a stressful event is more likely to illicit impaired functioning [31] and attempts to control unwanted events are not seen as a practical way to improve function [32, 33].

Conversely, average copers did not significantly differ in outcomes total scores from adaptive copers. Again, this may be explained by the degree of overlap in the levels of AS between the two groups, while the level of EA was significantly higher (Figure 1). The low levels of AS may have resulted in decreased need to control oneself and one's environment and greater level of flexibility and acceptance of disturbing experiences. Individuals less likely to avoid activities and more likely to persist in tasks have consistently been shown to have higher levels of physical and psychological functioning [34]. Another explanation is that AS has been shown to reflect one of the traits associated with obsessive personality among individuals with chronic pain [35]. Obsessive personality has been associated with psychopathology and health problems across a range of populations [29, 36–38]. Adaptive copers may also have lower levels of obsessive personality traits that would normally be associated with more negative outcomes.

Hence, average copers may not differ from dysfunctional copers due to the overlap in the level of EA and from adaptive copers due to the overlap in AS. This overlap provides a pseudocontrol condition among the subgroups, allowing the effect of AS or EA to be examined while controlling for the other. Bardeen et al. [13] demonstrated that though EA and AS predicted distress among individuals in the community with chronic pain, EA was a more important predictor of distress compared to AS. It is possible these results are limited due to the sample size. Perhaps a larger study may be able to observe a difference between the 3 groups. Lastly, the three groups did differ from each other on DASS-SF depression and anxiety subscales. As hypothesized, dysfunctional copers experienced the greatest distress based on higher endorsement for DASS-SF depression and anxiety subscales.

This study may be limited by its recruitment strategy which involved convenience sampling of individuals at one academic RA center. Additionally, information was not captured on those patients who refused to consent. This may also add to the bias of patient selection in the current study, thereby reducing the generalizability of the results to those individuals seen only in the community. The lack of information regarding comorbid issues and medication use limits the ability to control for these as cofounders. The cross-sectional nature of the study and the lack of a control group preclude conclusions regarding causation. The study's sole reliance on self-report measures may impact biases in reporting. It is also possible that patients responded to some of the items with a degree of bias such as their pain scores which required patients to recall symptoms from the past two weeks. Nonetheless, literature on bias related to pain recall has found that patients are still fairly accurate in recalling pain and pain characteristics even after 10 years; thus, we suspect the two-week recall would not overly bias patient reported pain [39].

Despite these limitations, the current study has important clinical and research implications. Much of the current data on AS and EA represents the chronic pain population; however, this study fills the gap in the effect of AS and EA among individuals with RA. The study demonstrates that the maladaptive effects of AS and EA are not limited to the chronic pain population but that they may also effect outcomes among those with RA. The current study also found that the effects of high EA and AS are not limited to mood but also relate to greater disability and lower QoL among those with RA.

Acceptance and commitment therapy has previously been shown to be effective in reducing overt dysfunctional behaviours such as escape avoidance behaviours and AS and encourage the acceptance of an individual's private experience [29]. Furthermore, McCracken and Keogh [40] found that the process of acceptance of pain, mindfulness, and values-based action reduce the extent to which AS interferes with functioning among individuals with chronic back pain. Future studies may need to explore these effects in the RA population.

In conclusion, the present study has important implications for the understanding of how, in a seemingly heterogeneous population of RA individuals with chronic pain, different subgroups may exist which predict mood, disability, and QoL. It may be important to identify patients that may be dysfunctional copers during the management of chronic pain among RA patients, since addressing these avoidant behaviours and the ability to experience distressing bodily sensation with patients may prove useful in helping to manage their pain. Acceptance based interventions that target EA by helping to develop more adaptive response styles and tolerance to undesirable experiences may help to improve outcomes among these individuals.

## Figures and Tables

**Table 1:** Demographic and clinical characteristics of study population and participants aggregated into the three cluster subgroups.

	Combined sample	Cluster 1	Cluster 2	Cluster 3	*p* (among clusters)
*N*	223	78	81	64	
Mean age (SD)	57.8 (14.8)	55.1 (13.0)	58.6 (16.2)	60.1 (14.5)	0.11
Sex (M%)	24.3	20.8	28.4	23.4	0.52
Relationship status (%)					
Single	10.0	9.3	7.5	14.1	0.29
Married or in a serious relationship	75.3	77.3	81.3	65.6	
Divorced, separated, or widowed	14.6	13.3	11.3	20.3	
Current employment (%)					
Employed	48.9	62.5	41.9	38.0	0.02
Unemployed	50.5	37.5	58.1	60.0	
Pain duration (%)	14.3 (10.8)	14.6 (10.5)	15.9 (12.7)	11.7 (7.7)	0.08
Average pain intensity (SD)	3.8 (2.2)	3.3 (2.4)	4.0 (2.1)	4.1 (2.1)	0.77
AAQ	32.2 (7.1)	24.5 (3.8)	35.4 (3.6)	37.5 (5.2)	<0.0001
ASI	15.0 (10.3)	8.6 (5.8)	11.2 (4.8)	27.8 (8.2)	<0.0001

SD: standard deviation; AAQ: Acceptance and Action Questionnaire; ASI: Anxiety Sensitivity Index.

**Table 2:** Mean values (standard deviation) in mood, disability, and quality of life among the three cluster subgroups.

	Cluster 1^a^	Cluster 2^b^	Cluster 3^c^	*p* (among clusters)
DASS-SF total	13.53 (11.98)^bc^	22.97 (18.20)^a^	29.30 (20.75)^a^	<0.001
DASS-SF depression	3.90 (3.76)^bc^	7.30 (6.80)^ac^	9.73 (7.60)^ab^	<0.001
DASS-SF anxiety	5.46 (5.31)^bc^	8.77 (7.08)^ac^	11.72 (9.00)^ab^	<0.001
DASS-SF stress	4.46 (4.49)^bc^	7.56 (5.46)^a^	9.72 (7.78)^a^	<0.001
HAQ Total	0.81 (0.71)^bc^	1.05 (0.73)^a^	1.18 (0.75)^a^	0.01
SF-36 total	98.9 (6.0)^c^	98.4 (6.6)	95.6 (6.8)^a^	0.04

Note: each superscript letter denotes outcomes whose column proportions differ significantly from that specific cluster subgroup at the 0.05 level. DASS-SF: Depression Anxiety Stress Scales-Short Form; HAQ: Health Assessment Questionnaire; SF-36: 36-Item Short Form Health Survey.
